# Towards a global plastics treaty

**DOI:** 10.2471/BLT.23.021223

**Published:** 2023-12-01

## Abstract

The passing of an effective global plastics treaty slated for 2024 would have major implications for public health. Gary Humphreys reports.

Hugh Shim knows what failure looks like. It goes floating past his office every day in the form of the plastic bottles, food containers and shopping bags that end up in Montego Bay, Jamaica. “I look out onto one of six estuaries that feed the bay,” explains the executive director of the Montego Bay Marine Park Trust. “The worst are the North and the South gullies.”

The failure is not the pollution itself, of course, but the multiple, and only partially effective initiatives to reduce it that have been implemented in Jamaica in recent years, several of them run by the park trust itself. “We have seen a lot of public clean-up campaigns over the years, including things we have done ourselves in terms of community- and school-led clean-up programmes, but the plastic keeps coming,” Shim says.

Most recently, the government of Jamaica entered a three-country initiative, along with Colombia and Panama, to combat plastic pollution. The initiative is being led by the United Nations Environment Programme (UNEP) with funding from the Global Environment Facility (GEF), a global trust fund investing in sustainability projects. According to Leah Karrer, a senior environmental specialist in the GEF secretariat, the three-country initiative aims to reach its stated goals by mainstreaming circularity at city-level.

Countries worldwide are struggling with similar challenges, and doing so against the backdrop of ballooning plastic production. The Organisation for Economic Co-operation and Development estimates that 460 million tonnes of plastic are manufactured each year worldwide, up from around 230 million tonnes in 2000. Based on current trends, global production will nearly triple by 2060, reaching around 1.2 billion tonnes.

According to Sheila Aggarwal-Khan, director of UNEP’s Industry and Economy Division, the bulk of that production, once used, eventually leaks into the environment. “Only around 9% of today’s production is recycled back into new products,” she says, pointing out that the term ‘recycling’ is itself misleading, with its suggestion of perpetuity; plastic can only be ‘recycled’ a few times, and each time to a product of lower quality.

While improperly discarded plastic certainly causes damage to the environment and is of significant concern to animal and human health, health risks exist at all stages of the plastics lifecycle, from production, use, recycling and eventual disposal, as well as from legacy plastics in the environment.

“At the plastics manufacturing end, there is growing evidence regarding the negative health impacts of some of the chemicals used,” explains Lesley Onyon, head of the Chemical Safety and Health unit at WHO.

“The treaty should support […] reuse, refill, and repair.”

Sheila Aggarwal-Khan

One example is bisphenol A (BPA), an important component in the production of polycarbonate plastics used in a wide range of consumer products, from food and drink containers to medical products. Numerous laboratory studies have shown that BPA can interfere with hormones in the endocrine system, leading to reproductive and developmental problems in humans and wildlife.

Phthalates are also of concern – a group of chemicals used to soften and increase the flexibility of plastics, some of which may be associated with reproductive and developmental issues as well as cancers.

Efforts to address the plastics challenge date back to the 1970s when a "Reduce, Reuse, and Recycle" mantra was first formulated. However, according to Karrer the focus has tended to be on the last ‘R’, putting most of the responsibility for dealing with plastic on consumers, while manufacturers continue to produce largely unrecyclable, unrepairable and rapidly obsolete products.

Karrer argues that reducing plastic pollution requires a shift to circularity, defined as minimizing waste and environmental impact throughout the entire lifecycle of plastic materials, stressing that circularity begins with designing products that use less plastic, can be re-used and repaired, and finding alternatives to plastics where feasible.

Compelling on paper, in the real world, circularity initiatives have been few and far between. “There have been some attempts to encourage reuse, one being the Algramo initiative originally launched in Chile,” says Karrer, naming a project that allows consumers to refill daily necessities such as shampoo, washing-up liquid and detergent, through Algramo vending machines.

According to Onyon, the health sector (a major consumer of single-use plastics in products ranging from syringes and intravenous bags to catheters, test kits, and gloves) is looking at alternatives to plastic. These include nitrile, neoprene and polyurethane in examination gloves rather than polyvinyl chloride, and using biobased raw materials in making medical textiles. Another product attracting attention is the bioplastic polyhydroxyalkanoates (PHA), an alternative to traditional fossil fuel-based plastic that is biodegradable across a range of natural environments.

While supportive of innovation, Aggarwal-Khan stresses the need to restrict the flow of unnecessary, problematic plastics and plastics that are not being recycled. “While designing products that can be reused many times, and their material recycled or composted has the potential to address part of the problem, curbing production of polymers that are not recyclable or not being recycled in practice is essential,” she says.

Like her peers in other agencies, Aggarwal-Khan is hopeful that a reduction in the output of such polymers will be one of the outcomes of a new global plastics treaty which is currently under discussion and is scheduled for completion in 2024.

First called for in a resolution passed by the United Nations Environment Assembly in March 2022, the potential contents of the treaty have since been debated in three sessions of the Intergovernmental Negotiating Committee responsible for developing the instrument, the last of which took place in November at UNEP Headquarters in Nairobi, Kenya.

What form the eventual treaty might take remains to be seen. However, a 31-page "zero draft" version that was published by the chair of the intergovernmental negotiating committee in September, and considered by delegates at the November meeting, may offer some indication.

Among the eye-catching optional propositions in the draft are those containing language relating to reductions in plastics production, as well as for the phase-out and eventual elimination of certain polymers and chemicals of concern.

“Reducing plastic pollution requires a shift to circularity.”

Leah Karrer

There is also language addressing producer responsibility, which calls for corporations being accountable for footing the bill for clean-up and recycling (the 'polluter pays' principal), along with proposals for possible financing mechanisms to accomplish that aim.

Aggarwal-Khan believes that to be effective, the instrument developed needs to drive comprehensive reform of the way we make and use plastic, not just focus on disposal. “The treaty should support the development of systems needed for reuse, refill, and repair, while also ensuring that waste management and recycling are on a par with the volume of waste created,” she says.

While stressing the need for robust governance, neither Aggarwal-Khan nor Karrer believe that plastics reduction or elimination will be possible unless viable alternatives are found; and both underline the importance of requiring governments to put in place policy incentives that encourage innovation and enable a market to flourish for safer alternatives.

Karrer believes that the private sector will have an important role to play in developing those alternatives, a view shared by Jayne Paramor, a strategic technical manager at the Waste and Resources Action Programme (WRAP), a nongovernmental organization committed to environmental causes, with considerable experience with private sector engagement and national-level plastics treaties.

The treaty will need to engage companies for whom plastics are immensely profitable and policy-makers concerned about the impact that transitioning to plastic alternatives may have on their economies.

On the corporate front, Paramor is optimistic. “I sit on a lot of panels, and corporate representatives are increasingly present,” she says. “They know this needs fixing.” In Paramor’s assessment, one of the drivers of the change in corporate attitudes is the increasing focus on the health harms associated with plastics. “Consideration of the impact of plastics on human health is really helping to drive the discussion,” she says.

With regard to potential government reluctance to embrace change, both Karrer and Aggarwal-Khan believe obstacles can be overcome if adequate attention is paid to achieving an equitable transition. “There is going to be a need for solidarity, support and funding,” says Aggarwal-Khan, underlining the importance of keeping people at the centre of the reform process, and making the most of opportunities to deliver new jobs and capture planet-friendly economic and health gains.

Hugh Shim also believes people should be central to reform, notably in their attitudes to product consumption. “I am from the days when you used to reuse and fix things,” he says. “If something was wrong with your shoe, you put a nail in the bottom. That doesn’t happen anymore. Now you throw the shoe away and it ends up in the bay. That needs to change too.”

**Figure:**
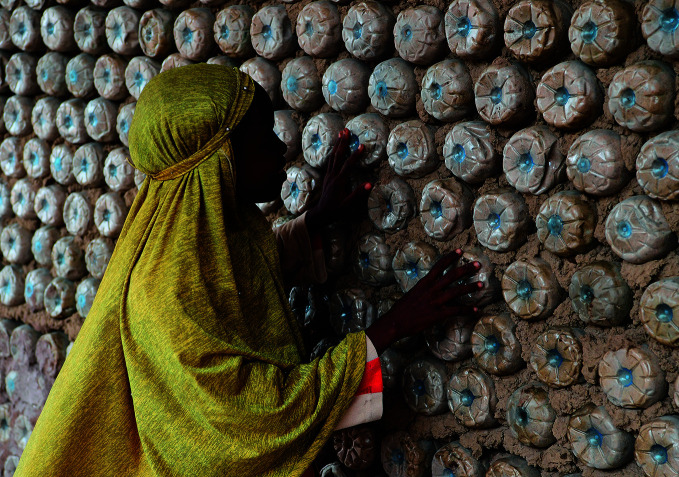
A schoolgirl in Ndjamena, Chad, examines a wall in her classroom made of plastic bottles filled with sand.

UNICEF/Frank Dejongh

**Figure:**
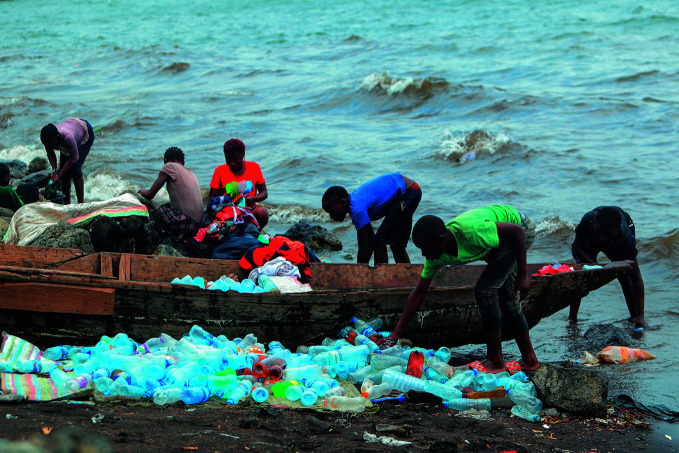
Cleaning bottles for resale in lake Kivu in the Democratic Republic of the Congo.

UNICEF/Sibylle Desjardins

